# Characterization of Genes Related to Intramuscular Fat Deposition in Muscles of Piglets Under Cold Exposure by Whole Transcriptome Sequencing

**DOI:** 10.3390/cimb48050463

**Published:** 2026-04-29

**Authors:** Fang Wang, Liang Wang, Zhenhua Guo, Hong Ma, Bo Fu, Dongjie Zhang, Di Liu

**Affiliations:** 1 Heilongjiang Academy of Agricultural Sciences Postdoctoral Programme, Harbin 150086, China; 2 Key Laboratory of Combining Farming and Animal Husbandry, Ministry of Agriculture, Animal Husbandry Research Institute, Heilongjiang Academy of Agricultural Sciences, Harbin 150086, China

**Keywords:** longissimus dorsi muscle, myosteatosis, pig, RNA, whole transcriptome sequencing

## Abstract

Background/Objectives: Understanding the regulatory mechanisms of intramuscular fat accumulation is crucial for maintaining skeletal muscle function and treating muscle-related diseases. It is known that cold exposure can lead to fat deposition in the muscles of mice and pigs. However, so far, there is very limited knowledge about the factors influencing its formation under cold exposure conditions. This study used piglets as an animal model to investigate intramuscular fat accumulation under cold exposure. Methods: Six piglets were exposed to 10 °C, and six piglets were exposed to 25 °C. A whole transcriptome joint analysis was performed on the longissimus dorsi muscle of three piglets randomly selected from each group. Results: No fever or cough symptoms were observed in all experimental groups, and the cold exposure vs. control groups’ RNA data were compared. The study identified 705 differentially expressed messenger RNAs, 87 long non-coding RNAs, 57 microRNAs, and 236 circular RNAs. *CD36 Molecule* (*CD36 Blood Group*) (*CD36*) was upregulated, while *adiponectin* (*ADIPOQ*) was downregulated. Conclusion: We established a competing endogenous RNA network centered around *CD36*, *Protein Phosphatase 1 Regulatory Subunit 3G* (*PPP1R3G*) and *ADIPOQ* for intramuscular fat accumulation by using a pig model exposed to a cold temperature. This study provides important references for further understanding the regulatory mechanism of intramuscular fat.

## 1. Introduction

Deep research of the molecular mechanisms underlying fat deposition in porcine muscle is fundamentally important for precisely improving pork quality, increasing the economic efficiency of pig production, supporting animal breeding and basic molecular biology research, and providing medical insights for human metabolic diseases. Intramuscular fat (IMF) is the key determinant of pork palatability, principally enhancing tenderness, flavor, juiciness, and marbling [1,2,3,4]. Longissimus dorsi is the most valuable and market-preferred region of the pig carcass. As a major skeletal muscle and source of lean meat, its efficiency of fat deposition directly affects the balance between lean yield and meat quality, and it is the industry-recognized standard site for measuring intramuscular fat content [5,6]. Dominated by fast-twitch (white) fibers, the longissimus dorsi is a prototypical skeletal muscle; its metabolic patterns and lipid deposition characteristics best reflect the physiological state of muscle, so findings can be extrapolated to most other skeletal muscles. During cold acclimation, lipid deposition responses in the longissimus dorsi are pronounced, facilitating the identification of key regulatory genes and pathways [7,8,9].

In general, pigs share similarities in physiologic, immunologic and genetic characteristics with humans [10]. Pigs as an animal model to study intramuscular fat accumulation can provide a theoretical basis for human muscle-related diseases. Cold exposure plays a role in lipid metabolism in many ways, such as increased conversion of cholesterol to bile acids [11] and decreased triglyceride levels [12]. Cold exposure has been shown to induce intramuscular fat accumulation in the skeletal muscles of pigs and mice [13,14]. However, the molecular mechanisms of intramuscular fat deposition under cold exposure remain unclear.

In the field of human medicine, Intramuscular fat accumulation (fat infiltration in muscle, intramuscular fat deposition, and myosteatosis) directly affects muscle function and harms human health [15,16,17]. Skeletal muscle accounts for approximately 40% of total body weight [15], and fat constitutes about 30% of body weight [18]. Abnormal deposition of fat in non-adipose tissues can lead to conditions such as fatty liver [19] and subcutaneous lipomas [20]. If fat accumulates in muscle tissue, it results in intramuscular fat accumulation [15]. Currently, the use of a common animal model involves injecting glycerol into the skeletal muscle of mice to induce muscle damage and regeneration and examining intramuscular fat accumulation [21].

Competing endogenous RNAs (ceRNAs) are endogenous RNA molecules that share common microRNA (miRNA) binding sites and regulate one another’s expression by competitively binding the same miRNAs. Intramuscular fat accumulation is a highly complex regulatory process involving numerous cellular signaling pathways, messenger RNA (mRNA), long non-coding RNAs (lncRNA), circular RNA (circRNA), and microRNA (miRNA). By constructing ceRNA networks, disparate differentially expressed mRNAs and differentially expressed lncRNAs/circRNAs can be linked through miRNAs to form regulatory networks, which helps explain the molecular basis of abnormal differential gene expression, identify core regulatory axes, and deepen the study of mechanisms underlying intramuscular fat deposition [22]. There have been reports analyzing the ceRNA regulatory network after pigs were injected with vitamin A [2]. This study established the ceRNA regulatory network of intramuscular fat accumulation in pigs after cold exposure through whole transcriptome sequencing, identifying the key genes *CD36 Molecule* (*CD36 Blood Group*) (*CD36*) and *adiponectin (ADIPOQ*). The findings may contribute to understanding the molecular mechanism of intramuscular fat deposition, as well as providing insights for maintaining the function of skeletal muscle and treating muscle-related diseases [15].

## 2. Materials and Methods

### 2.1. Animals

This research was approved by the Committee for Animal Welfare at the Institute of Animal Husbandry, Heilongjiang Academy of Agricultural Sciences (HAAS) (No. NKYXMS-20240517, 17 May 2024), in accordance with the Laboratory Animal Guidelines for Ethical Review of Animal Welfare (GB/T 35892-2018) [23]. After the experimental pigs were confirmed to be fully anesthetized and unconscious, the common carotid artery was immediately isolated and severed to minimize potential suffering.

The experiments were conducted at the HAAS experimental farm; the thermoneutral zone for 5–6-week-old piglets is approximately 22–26 °C. The information on piglets is listed in Supplementary Table S1. Yorkshire piglets were co-housed for two weeks before the commencement of the trial. The piglets were held in an artificial climate chamber with controlled temperature and relative humidity during the entire experimental process. Twelve piglets (weighing 11–16 kg, 5–6 weeks) were randomly divided into a cold exposure group (six piglets at 10 °C) and a control group (six piglets at 25 °C) [24]. The experimental process does not restrict diet and water intake. The temperature change information of the artificial climate chamber and feedstuff content are listed in Supplementary Table S2. After 63 h [25], three piglets that showed no signs of fever or cough and were apparently healthy were selected from each group and slaughtered in accordance with animal welfare guidelines. The longissimus dorsi muscles were collected and immediately placed in liquid nitrogen for preservation for subsequent experiments [5,6]. The experimental sequence involved one piglet from each of the cold exposure and control groups being processed in turn. Considering that cold exposure under artificial climate chamber conditions may cause stress and symptoms such as cough and dysentery in piglets, this study prepared double experimental subjects to avoid interference with experimental results due to illness of the subjects. If all piglets were healthy after the experiment, only half of them were selected for the experiment.

### 2.2. Longissimus Dorsi Muscle Hematoxylin-Eosin Staining

The Longissimus dorsi muscle sample is quickly placed in 10% acetone and fixed for 24 h to ensure that the tissue block does not float above the liquid. Dehydrate using an alcohol gradient, followed by transparency with xylene. After embedding in paraffin, slice perpendicular to the fiber direction to a thickness of 3 µm. Then hematoxylin eosin (HE) staining is performed. Sections were stained with hematoxylin solution for 5 min, rinsed with running water for 5 min, treated with 1% hydrochloric acid ethanol for 30 s, and then washed with water. Stain with 0.5% eosin solution for 2 min, wash with distilled water and ethanol, and seal with gum. Record scanned photos at 200× and 400× magnification, respectively.

### 2.3. Measurement of Intramuscular Fat Content

The longissimus dorsi muscle samples were minced and placed into a filter paper thimble. After weighing, they were put into a Soxhlet extractor (Pony Testing Technologies Co., Ltd., Harbin, China). After adding petroleum ether, the mixture was heated and refluxed 25 times, after which the heating was stopped. The samples were dried and weighed to obtain the intramuscular fat content [5]. Student’s *t*-test was used to analyze the date, and the results were shown as mean ± SD (Standard Deviation). *p* < 0.05 was defined as significant; *p* < 0.01 was defined as very significant. The plot was created by GraphPad Prism software (version 8.0.2, San Diego, CA, USA).

### 2.4. Whole Transcriptome Sequencing

Samples of the longissimus dorsi muscle were removed from the liquid nitrogen and mailed on dry ice to Beijing Biomarker Technologies Co., Ltd., Beijing, China (https://www.bmkgene.com) for whole transcriptome joint analysis. The extracted total RNA was tested for integrity using the Agilent 2100 BioAnalyzer (Agilent Technologies Inc., Santa Clara, CA, USA) and LabChip GX Touch (PerkinElmer Instruments Co. Ltd., Hopkinton, CT, USA). After the construction of the RNA library, the Qsep400 high-throughput analysis system (BiOptic Inc., Santa Clara, CA, USA) was employed to inspect the insert size of the library, acquiring raw data of the mRNA and non-coding RNA. Dispersion estimates and power calculations were conducted using the “BiocManager”, “BiocParallel”, “DESeq2”, “ggplot2”, and “reshape2” packages in R software (v4.5.3, R Foundation for Statistical Computing, Vienna, Austria). During the testing process, the experimental personnel were not aware of the group assignments for each sample. It was only during the data analysis that the sample groups were correlated with the test results.

### 2.5. Quantitative Real-Time PCR (qRT-PCR) Verify mRNA

Based on the results of mRNA sequencing, 8 genes were randomly chosen to verify the accuracy of the results. Furthermore, subsequent analysis indicated that *CD36* and *ADIPOQ* are very important target genes, and validation was also performed for these genes. Total RNA was extracted from muscle samples using TRIzol reagent (Invitrogen, Waltham, MA, USA, 15596026CN). cDNA was synthesized using a cDNA synthesis kit (Toyobo, Osaka, Japan, FSQ-301) through reverse transcription. qRT-PCR was performed using SYBR™ Green (Toyobo, Osaka, Japan, QPX-201). The primers for the 10 genes are detailed in Table 1.

### 2.6. RNA Principal Component Analysis and Differential Expression Analysis

Principal component analysis (PCA) was performed to verify the rationale of the grouping method used in this study. The analysis was conducted on the mRNA, lncRNA, miRNA, and circRNA from six samples to confirm that the data detected by the grouping method showed intergroup differences. Subsequently, the differential expression data of the four RNA types were visually represented using volcano plots, with the log _10_ fold change (FC) value set at ±0.6 (FC = ±4), the *p*-value for mRNA and lncRNA set at 0.05 (−log _10_ *p* = 1.3), and the alpha value for miRNA and circRNA set at 0.01 (−log _10_ *p* = 2). The RNAs involved in the ceRNA network identified in Section 2.6 were marked.

### 2.7. Differential Expression of RNA Correlation Analysis

To conduct a joint analysis of the targeting correlations of differentially expressed RNAs, focus was placed on identifying other differentially expressed RNAs that have targeting correlations with the core elements, such as mRNA and miRNA. Pairs that show differential correlations among the circRNA, lncRNA, miRNA, and mRNA were extracted. The data obtained from this analysis were visualized in Venn diagrams created with Biocloud (https://www.biocloud.net). The results of this analysis were subsequently used for the ceRNA network analysis.

### 2.8. Gene Ontology (GO) and Kyoto Encyclopedia of Genes and Genomes (KEGG) Enrichment Analysis

For the differentially expressed mRNAs that were upregulated and downregulated, GO annotation was performed separately for the three categories: Biological Process, Molecular Function, and Cellular Component. The analysis focused on the top ten terms with the highest gene ratio. Similarly, KEGG enrichment analysis was conducted for the upregulated and downregulated genes, focusing on the top 20 pathways with the highest gene ratio. Bubble charts and string diagrams were generated to visualize the enrichment results. The analysis results correspond to the ceRNA analysis in the next section.

### 2.9. Integrated ceRNA Regulatory Network

Based on the interactions and targeting correlations among the differentially expressed RNAs, the co-expressed parts that exist within the intersections of Venn diagrams were identified. The GO terms and KEGG pathways involved in fatty acid metabolism were compared and analyzed to ultimately determine the regulatory ceRNA network that controls intramuscular fat accumulation. This network included mRNA, lncRNA, miRNA, and circRNA. Normalizing the potentially controversial genes using the following formula:

$$Gene\text{\_}normal=\frac{Gene}{\sqrt{markA\;gene*markB\;gene}}$$

## 3. Results

### 3.1. Cold Exposure Leads to Intramuscular Fat Accumulation

**Figure 1:**
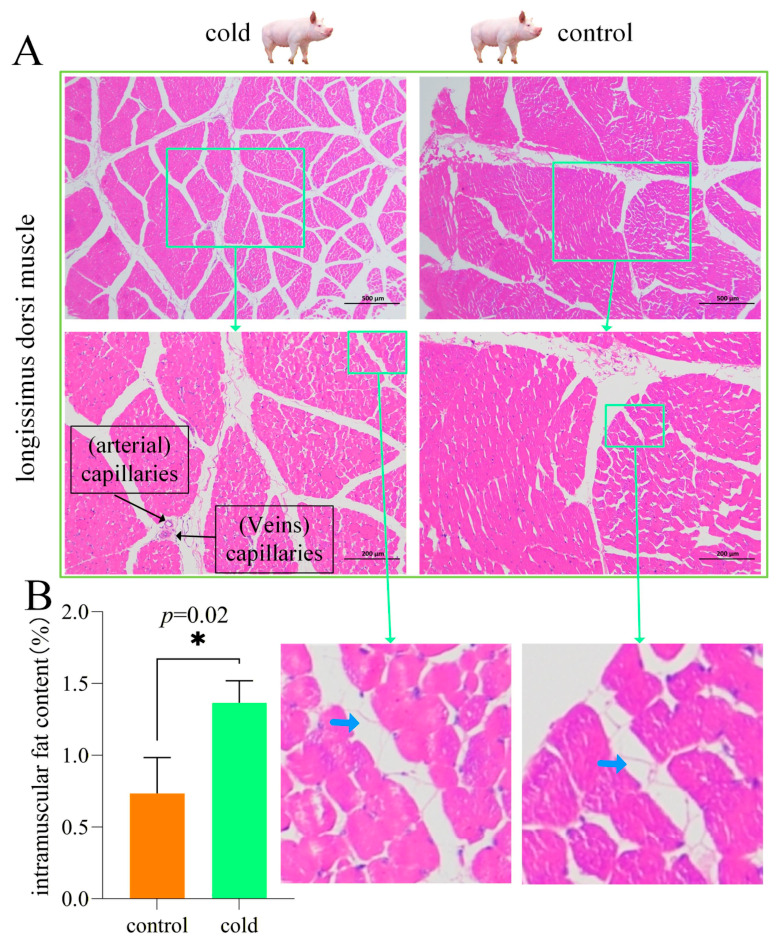
Intramuscular fat of the longissimus dorsi muscle. (**A**) Hematoxylin-eosin staining of muscle fibers and fiber bundles in the cold exposure group and control group. Muscle cell nuclei are blue and cytoplasm is red. The blank space between the fiber bundles represents the lost adipocytes, and the blue arrow indicates the adipocyte cell wall. The black bars in the picture represent 200 or 500 µm. The first line of the micrograph is magnified 200 times, and the second line is magnified 400 times. The green boxes and arrows represent enlarged views. (**B**) Intramuscular fat content of longissimus dorsi muscle. * indicates significant difference at *p* < 0.05.

The results of longissimus dorsi muscle HE staining demonstrate that the cold exposure group deposited more fat between the fiber bundles compared to the control group, accompanied by the proliferation of connective tissue (Figure 1A). Figure 1B shows a significant increase in intramuscular fat content after cold exposure (*p* < 0.01).

### 3.2. Cold Exposure Leads to Differential RNA Expression

**Figure 2:**
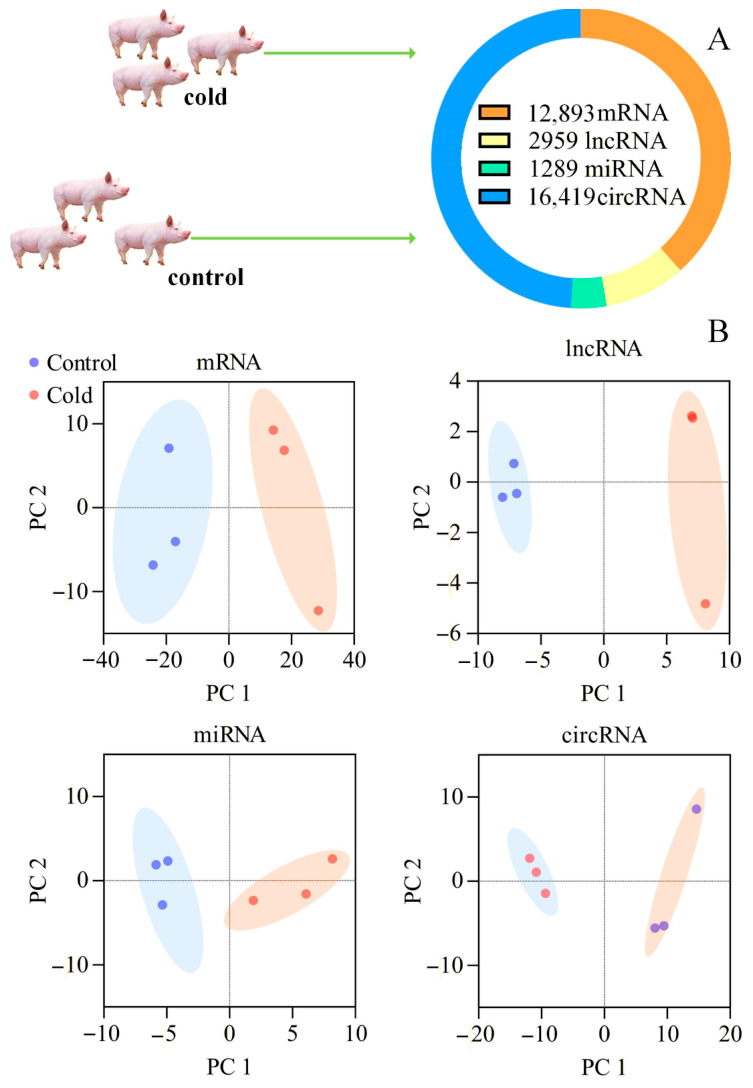
Results of whole transcriptome sequencing and principal component analysis. (**A**) Total amount of mRNA, lncRNA, miRNA, and circRNA from whole transcriptome sequencing. RNA was collected from cold-exposed piglets (10 °C) and control piglets (25 °C). (**B**) PCA of mRNA, lncRNA, miRNA, and circRNA.

**Figure 3:**
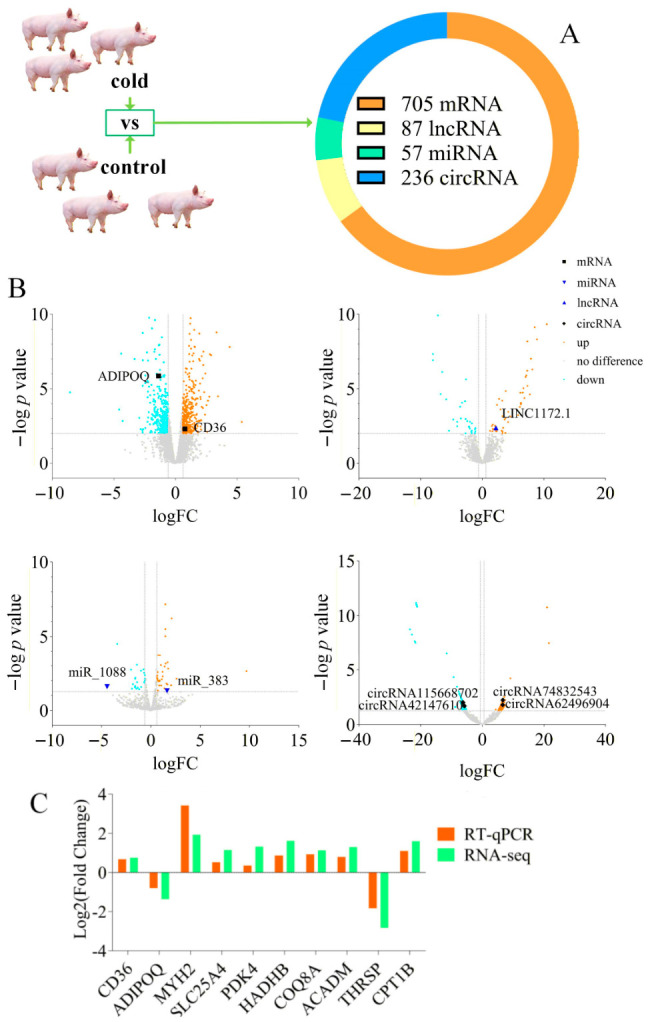
Differentially expressed RNA in the longissimus dorsi muscle. (**A**) Total amount of differentially expressed mRNA, lncRNA, miRNA, and circRNA from cold-exposed piglets (10 °C) and control piglets (25 °C). Green arrows indicate the workflow for identifying differentially expressed genes from pigs in the cold and control groups. (**B**) Volcano plots of differentially expressed mRNA, lncRNA, miRNA, and circRNA. The RNAs marked on the plots are involved in the ceRNA network regulation. (**C**) qRT-PCR results of 10 genes (cold exposure vs. control groups).

Neither the cold-exposure group piglets nor the control group piglets exhibited symptoms of fever or cough. Therefore, three piglets were randomly selected from each group for whole transcriptome sequencing. The results of dispersion estimates and power calculations are presented in Supplementary Figure S1, which evaluates the credibility of the data. Figure 2A shows the total amount of RNA detected. Figure 2B represents the PCA results for mRNA, lncRNA, miRNA, and circRNA, indicating that the data detected in the group experiment were clearly divided into two distinct groups. Figure 3A shows the differentially expressed RNAs detected. Up-regulated mRNA includes *Protein Phosphatase 1 Regulatory Subunit 3G* (*PPP1R3G*), *CD36*, *Contactin-Associated Protein Family Member 5* (*CNTNAP5*), etc. Down-regulated mRNA includes *TNF Alpha Induced Protein 6* (*TNFAIP6*), *Speckle Type BTB/POZ Protein Like* (*SPOPL*), *ADIPOQ,* etc. Figure 3B displays the volcano plots of the differential expression of mRNA, lncRNA, miRNA, and circRNA, with the RNAs marked on the graph being those included in the ceRNA network. The three most strongly downregulated mRNAs were *CD163 Molecule-Like 1* (*CD163L1*)*, Secreted Phosphoprotein 1* (*SPP1*)*, and Phosphatidylinositol Glycan Anchor Biosynthesis Class Z* (*PIGZ*); the three most strongly upregulated mRNAs were *Adenosine Monophosphate Deaminase 3* (*AMPD3*)*, CF Transmembrane Conductance Regulator* (*CFTR*)*, and Rab Interacting Lysosomal Protein* (*RILP*). However, no related ceRNA networks were found for these genes. The results of Figure 3C indicate that the qRT-PCR results are consistent with the mRNA sequencing results.

### 3.3. Targeting Correlations Between Differentially Expressed RNAs

**Figure 4:**
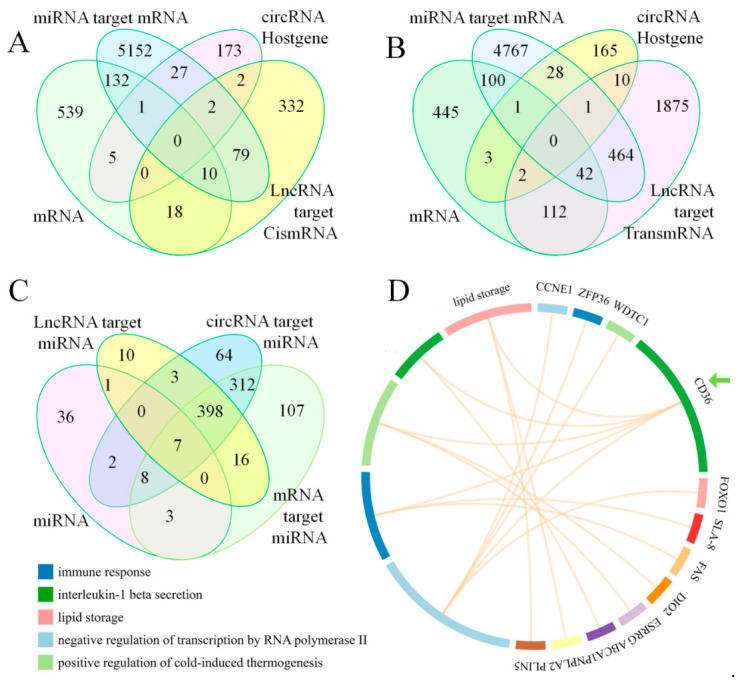
Venn diagrams of differentially expressed RNAs and Gene Ontology (GO) annotation of upregulated genes. (**A**) lncRNA targeting cis-mRNA. (**B**) lncRNA targeting trans-mRNA. (**C**) Venn diagram constructed using differentially expressed miRNAs as the core, combined with other differentially expressed RNAs that have targeting relationships. (**D**) Top 5 biological processes from the GO annotation of upregulated mRNAs.

Using differentially expressed mRNAs as the core, Figure 4A,B illustrate the targeting correlations with other differentially expressed RNAs. Figure 4A shows the lncRNA targeting cis-mRNA, while Figure 4B shows lncRNA targeting trans-mRNA. Centered on differentially expressed miRNAs, Figure 4C shows the target correlations with other RNAs. The overlapping parts among these three different RNAs were utilized to screen for the ceRNA network. These overlapping regions represent potential competitive binding interactions and are critical for revealing the molecular basis of aberrant gene expression and the core regulatory axis. In short, based on the Venn diagram, we used the mRNA as the core to search for related ceRNAs. The selection criteria were that mRNA and miRNA expression directions must be opposite, while mRNA and circRNA expression directions must be the same; under this premise, if a circRNA found can regulate the miRNA, it is included as a ceRNA. The same method was used to identify mRNA–lncRNA–circRNA regulatory networks.

### 3.4. GO and KEGG Enrichment Analysis

**Figure 5:**
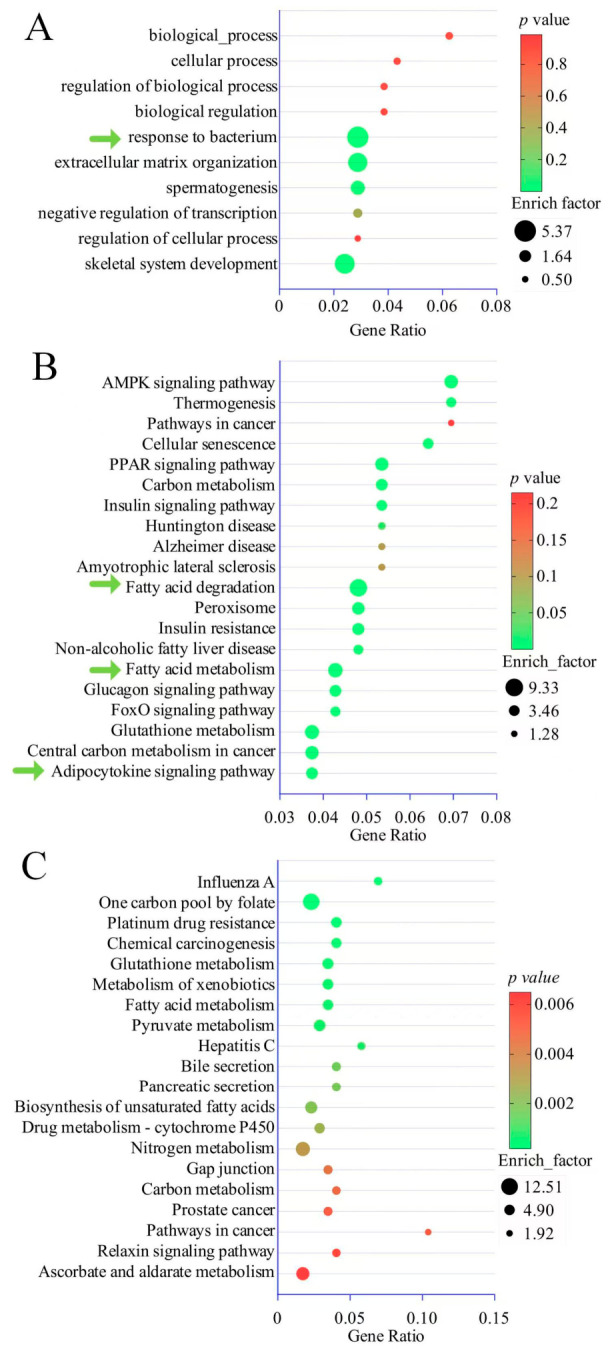
GO and KEGG enrichment analysis of differentially expressed RNAs. (**A**) Top 10 biological processes from the GO annotation of downregulated mRNAs. (**B**) Top 20 pathways from the KEGG enrichment analysis of upregulated mRNAs. The green arrows indicate pathways related to intramuscular fat accumulation or thermogenesis. (**C**) Top 20 pathways from the KEGG enrichment analysis of upregulated mRNAs.

The GO annotation system of the upregulated genes among the differentially expressed mRNAs revealed the upregulation of *CD36* promoted lipid storage and positive regulation of cold-induced thermogenesis (Figure 4D, green arrow). Figure 5A (green arrow) displays the downregulation annotation of mRNAs, where the expression of *ADIPOQ* decreased, reducing the response to bacteria. The KEGG enrichment analysis of differentially expressed upregulated mRNAs identified the adenosine monophosphate-activated protein kinase (AMPK) signaling pathway, the peroxisome proliferator-activated receptor (PPAR) signaling pathway, the insulin resistance and adipocytokine signaling pathway, fatty acid metabolism, and fatty acid degradation (Figure 5B). However, the top 20 pathways in terms of gene ratio from the KEGG analysis did not enrich pathways related to *ADIPOQ* (Figure 5C).

### 3.5. Construction of the ceRNA Network

**Figure 6:**
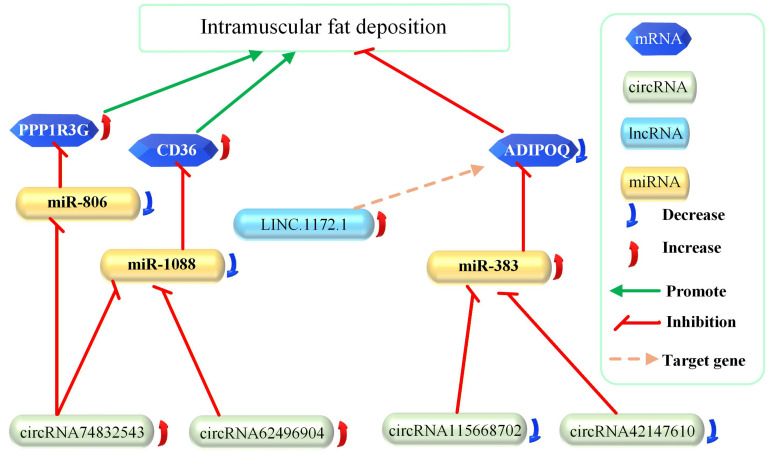
CeRNA network regulation of intramuscular fat accumulation. Intramuscular fat accumulation is positively correlated with *CD36* and negatively correlated with *ADIPOQ*. This also includes lncRNA, miRNA, and circRNA. Linc. 1172.1 is confirmed to be involved in the regulation of *ADIPOQ*, but the specific regulatory mechanism is uncertain.

After excluding the interactive targets among differentially expressed RNAs one by one and combining the comparative analysis of GO terms and KEGG pathways, the key genes identified in regulating intramuscular fat accumulation were the upregulated *CD36 and PPP1R3G* and the downregulated *ADIPOQ*. Figure 6 illustrates the regulatory ceRNA network, which includes mRNA, lncRNA, miRNA, and circRNA. The main purpose of this study was to identify ceRNA networks related to intramuscular fat deposition under cold exposure. We constructed ceRNA networks for all differential genes and obtained ceRNA networks centered on *CD36*, PPP1R3G, and *ADIPOQ*. Although *CD163L1*, *SPP1*, *PIGZ*, *AMPD3*, *CFTR*, and *RILP* showed the largest and most significant changes between the cold exposure and control groups, they did not form part of any ceRNA network and therefore were not selected.

Normalizing *ADIPOQ* with adipocyte marker genes (*FABP4*, *PLIN1*) using the following formula:

$$ADIPOQ\text{\_}normal=\frac{ADIPOQ}{\sqrt{FABP4*PLIN1}}$$

The results of *ADIPOQ* normalization are listed in Supplementary Table S1. Compared with the control group, the *ADIPOQ* of the cold treatment group was significantly reduced (*p* < 0.05).

## 4. Discussion

This study established a regulatory ceRNA network entirely in silico for intramuscular fat accumulation by using a pig model exposed to a cold temperature. The key genes involved are *CD36*, *PPP1R3G* and *ADIPOQ*.

### 4.1. Cold Exposure Promotes Intramuscular Fat Accumulation

The *UCP1* gene defect in pigs limits fat thermogenesis, making them highly sensitive to cold stimuli [26]. Intramuscular fat accumulation and muscle thermogenesis seem to be contradictory processes; the former involves energy storage, while the latter involves energy expenditure. However, these two processes occur simultaneously under cold exposure. Skeletal muscles maintain energy stability throughout the body as they account for almost half of body weight and a high rate of fuel consumption [15]. Studies by other teams have shown that some genes involved in white fat browning are highly expressed in skeletal muscle under cold exposure [27]. The fat deposited into the muscles is used to provide energy for heat production. Our study’s GO enrichment analysis revealed that upregulated genes were involved in lipid storage, cold-induced thermogenesis, and immune response (Figure 4D). Both lipid storage and immune response indicated fat infiltration in the muscle. Short-term cold exposure (4 °C for 3 d) in 10-week-old male C57BL6/J mice led to a significant increase in intramuscular fat deposition [13]. After cold exposure, the intramuscular fat content of Duroc × Landrace × Yorkshire boars increased from 12.15% to 13.12%, and there was a significant change in fatty acid composition [14]. The upregulated genes in our study were also enriched in fatty acid metabolism and fatty acid degradation (Figure 5B). Under cold conditions, high-feeding yaks can accumulate more intramuscular fat than low-feeding yaks [28]. All cold experimental animals in our study were increasing intramuscular fat content also.

### 4.2. Correlation Between Intramuscular Fat Accumulation and Muscle Thermogenesis

Under cold stimulation, pigs will produce heat by shivering thermogenesis and non-shivering thermogenesis of muscle. A study of humans showed that ten male subjects wearing light clothing were found to rely on intramuscular fat for the energy required during exercise under cold exposure conditions [29]. Nine young male participants, after undergoing a 5 min cold water immersion immediately following a training session, had exercise-induced infiltration of inflammatory cells and subcellular translocation of heat shock proteins in muscles [30]. Muscle tissue is capable not only of shivering thermogenesis but also of non-shivering thermogenesis activated under cold exposure through the mediation of sarcolipin (SLN). Mechanistically, SLN acts as a regulator of the muscle/sarcoplasmic reticulum Ca^2+^-ATPase (SERCA) pump, interacting with SERCA in the presence of Ca^2+^ and activating non-shivering thermogenesis in muscle through Ca^2+^ futile cycling [31]. The influx of fatty acids into muscle serves as a substrate for cold-induced thermogenesis.

Under cold stimulation, the non-shivering thermogenesis mediated by SLN in skeletal muscle is compensatorily activated, suggesting a synergistic and interactive correlation between the thermogenic functions of brown adipose tissue and skeletal muscle, working together to adapt to changes in body temperature [32,33]. However, how adipose tissue coordinates the non-shivering thermogenesis process in muscle tissue is still unclear. Nevertheless, exosomes secreted by adipocytes are likely involved in this process [34,35]. MiR-27a from 3T3-L1 adipocyte-derived exosomes has been shown to inhibit insulin signaling in C2C12 cells via the PPARγ pathway [36,37]. The upregulated genes in our study were also enriched in the PPAR signaling pathway, AMPK signaling pathway and fatty acid degradation (Figure 5B). Given that AMPK is generally associated with the promotion of catabolic processes, other studies have also reported that cold exposure promotes the activation of the AMPK pathway in porcine muscle tissue to generate heat [38]. Paradoxically, cold exposure also promotes lipid deposition; thus, the two aforementioned processes, muscle thermogenesis and intramuscular fat accumulation, occur simultaneously under cold stress. We hypothesize that under cold exposure, skeletal muscle undergoes rapid physiological adaptation to support sustained non-shivering thermogenesis. This process requires a substantial amount of energy, leading to a continuous transport of fatty acids to the muscles to be decomposed for heat production, while excess lipids are stored. The fact that does not restrict diet and water intake in this study likely facilitated this process. Therefore, the observed intramuscular fat deposition is not a sample storage of excess energy but rather a complex energy storage mechanism driven by the catabolic demands of the AMPK pathway and fatty acid degradation-induced thermogenesis. Cold exposure typically induces hyperphagia in pigs, and elevated dietary lipid flux alone can drive IMF accumulation. This study did not restrict diet, which may have driven this process.

### 4.3. CD36, PPP1R3G and Intramuscular Fat Accumulation

*CD36* codes for a transmembrane protein involved in the transport of long-chain fatty acids across the cell membrane [39]. The expression level of *CD36* is positively correlated with intramuscular fat accumulation [40]. In mice, oral administration of erucic acid for eight consecutive weeks activated the PPARγ-FABP4/CD36 pathway; specifically, erucic acid increased CD36, promoting intramuscular fat accumulation [40]. Mice deficient in CD36 exhibited significantly lower levels of intramuscular fat accumulation, obesity, and oxidative stress compared to a control group [41]. Our study found that the expression level of *CD36* increased in the cold-exposure group (Figure 3B), and the GO enrichment analysis revealed that the upregulation of *CD36* in the Biological Process promoted lipid storage (Figure 4D), consistent with previous research findings. Adding retinoic acid to cattle feed can increase the production of marbled beef [42]. It is a positive correlation between CD36 and intramuscular fat accumulation, which is consistent with the conclusion of pigs.

The main function of *PPP1R3G* is to regulate the deposition and transformation of glycogen. PPP1R3G mediates glycogen synthesis in the liver and further regulates blood glucose level stability [43]. In mice exposed to a high-fat diet, serum levels of *PPP1R3G* were significantly decreased [44]. Mice lacking *PPP1R3G* are unable to deposit glycogen and do not gain weight even when faced with a high-fat diet [45]. So far, there have been no reports known to us on the regulation of intramuscular fat accumulation by *PPP1R3G*. This study reveals that an increase in *PPP1R3G* expression may have a positively correlated effect on intramuscular fat accumulation.

### 4.4. ADIPOQ Decrease and Intramuscular Fat Accumulation

Adiponectin coded by *ADIPOQ* is a protein secreted by white adipose tissue that plays a role in the regulation of glucose and lipid metabolism [46]. Overexpression of human *ADIPOQ* in transgenic mice resulted in suppression of body fat accumulation [47]. The *ADIPOQ* gene is a key gene in the regulation of intramuscular fat accumulation [15,48]. The correlation between *ADIPOQ* and intramuscular fat accumulation is very complex and remains uncertain [49].

There is also a seemingly contradictory issue that ADIPOQ is specifically secreted by adipocytes. If the IMF increases, it means that the mRNA of *ADIPOQ* will increase, while this study detected a decrease in *ADIPOQ* expression, and confirmed through normalization that mRNA is indeed significantly reduced (Supplementary Table S3). The reason for this may be due to the activation of the FOXO and PPAR signaling pathways after cold exposure (Figure 5B), with an increase in *FOXO1* expression (log _10_ FC = 0.68) and a decrease in *PPAR γ* expression (log _10_ FC = −0.77). This indicates that cold exposure activates the sympathetic nervous system, which in turn activates the FOXO signaling pathway. The activated *FOXO1* acts as a transcriptional repressor, directly inhibiting the transcription of *PPAR γ*. Research has shown that the PPAR signaling pathway can promote intramuscular fat deposition [50]. Despite the overall activation of the PPAR signaling pathway after cold exposure, which promotes fatty acid uptake and lipid droplet synthesis, leading to increased intramuscular fat deposition, the decreased expression of *PPAR γ*, as the core transcriptional activator of *ADIPOQ* [51], directly results in a significant decrease in that mRNA expression level.

Continuous monitoring of the longest dorsal muscle in sheep at different growth stages revealed that the *ADIPOQ* gene was negatively correlated with intramuscular fat accumulation [52]. The knockdown of goat *ADIPOQ* accelerated intramuscular fat accumulation and promoted the differentiation of intramuscular preadipocytes [53]. In a study of 22 elderly individuals (nine men, 13 women), *ADIPOQ* inhibited intramuscular fat accumulation and positively affected insulin sensitivity [54].

However, contrary results may occur in cattle. Single-nucleus RNA sequencing of Japanese black cattle muscle found that the pectoralis muscle (brisket) contains a higher proportion of intramuscular fat compared to the sternocleidomastoid muscle (neck), but the expression of *ADIPOQ* in the brisket is significantly higher than in the neck [55]. In bulls and steers, *ADIPOQ* correlated positively with intramuscular fat accumulation and negatively with the ribeye area [56]. In mice, the expression of *ADIPOQ* after intramuscular injection of glycerol showed a significant positive correlation with intramuscular fat accumulation [48]. The same result was obtained in *ADIPOQ*-Cre mice after intramuscular injection of glycerol [57].

In this study, the expression level of *ADIPOQ* decreased after cold exposure, which was negatively correlated with intramuscular fat accumulation. This expression pattern was similar to human intramuscular fat accumulation but differed from that observed in mice and cattle models. Compared with other livestock, pigs have a shorter gestation period, and their genomes are also easier to edit. In the future, the molecular mechanism by which *ADIPOQ* regulates intramuscular fat deposition can be studied through gene-edited pigs, providing clinical evidence for understanding human diseases.

### 4.5. Signal Pathways and Intramuscular Fat Accumulation

The signaling pathways that regulate intramuscular fat accumulation mainly include the AMPK signaling pathway [58], the mitogen-activated protein kinase signaling pathway [59,60,61], the PPAR signaling pathway [2,57], and the cyclic AMP-protein kinase A signaling pathway [62]. Our study also showed the enrichment of the AMPK and PPAR signaling pathways (Figure 5B). Metabolites from fat can disrupt the insulin signaling pathway [17]. Muscle dysfunction is closely related to aging, sarcopenia, diabetes, and obesity [15,16]. The upregulated genes in our study also enriched the insulin signaling pathway (Figure 5B). In addition, the most highly upregulated mRNA, the *CFTR* gene, participates in fatty acid and triglyceride synthesis and promotes fat deposition [63]. In *CFTR* knockout mouse studies, even under normal feeding conditions, mice lacking *CFTR* showed weight loss [64]. *AMPD3* promotes deposition of fatty acids from the blood into muscle, increasing intramuscular fat content in sheep, goats, and pigs [46,65,66].

Giving vitamin A to piglets can increase intramuscular fat accumulation in the longissimus dorsi muscle, and a potential ceRNA regulatory network has been proposed that controls nine genes, including *IRF1* [2]. By comparing Duroc pigs with high and low intramuscular fat, many differentially expressed circRNAs were identified, and their host genes (*TTC7B*, *PSD3*, *GOT2*) are related to fat metabolism [67]. However, the ceRNA regulatory network obtained from cold exposure in our study is centered around the regulation of *CD36* and *ADIPOQ*. Tianfu black rabbits fed a high-fat diet showed increased expression of *miR383*, which promotes intramuscular fat accumulation [68]. This is consistent with the results of our study.

## 5. Limitation

This study analyzed six samples, and the experimental sample size is relatively small, which limits the direct recommendation of animal models for human diseases. The dispersion plot of mRNA estimates shows a decreasing trend from top left to bottom right, with a flat tail that conforms to the RNA seq data pattern. Power calculations of mRNA indicate that the screened differentially expressed genes are relatively few, which may result in false negatives (Supplementary Figure S1), so the FC set for RNA in this study is ±4 (log _10_ FC = ±0.6). A previous review discussed the effects of cold exposure time on adipocytes in pigs [69]. Known studies range from 4 h to 15 days, but this study suggests that 63 h at 10 °C is an acceptable time. However, it is uncertain whether this time is the most suitable detection time and has certain limitations.

Furthermore, it should be noted that the transcriptomic changes identified in this study reflect the comprehensive gene expression profile of the entire skeletal muscle tissue, encompassing both myocytes and infiltrating adipocytes. Although histological analysis demonstrated a visible increase in adipocyte presence, their nuclei are relatively small; thus, their proportionate contribution to the bulk tissue RNA pool is considered minimal compared to that of the predominant myonuclei. Recent research emphasizes that intramuscular fat deposition is governed by a dynamic, co-regulated network involving crosstalk between myocytes and adipocytes [70]. Consequently, our bulk RNA sequencing approach captures the holistic, tissue-level transcriptomic shifts associated with cold-induced intramuscular fat deposition, rather than resolving cell-type-specific regulatory mechanisms [70]. This study did not involve single-cell sequencing, and whether the observed upregulation of *CD36* and downregulation of *ADIPOQ* reflect genuine transcriptional regulation has limited credibility, as the results may simply reflect altered cellular composition in the sampled tissue. This will be a direction for our future research.

## 6. Conclusions

After cold exposure, pigs exhibited differential expression of mRNA, lncRNA, miRNA, and circRNA in the longissimus dorsi muscle. We constructed a ceRNA network entirely in silico centered around *CD36*, *PPP1R3G* and *ADIPOQ* for intramuscular fat accumulation. *CD36* and *PPP1R3G* are upregulated, while *ADIPOQ* is downregulated, with miR-1088, miR-383, circRNA74832543, circRNA62496904, circRNA115668702, and circRNA42147610 involved in this process. The ceRNA regulatory network of intramuscular fat accumulation obtained through cold exposure is entirely different from that achieved by vitamin A injection. It was discovered that the increase in *PPP1R3G* expression is positively correlated with intramuscular fat accumulation. The information of this ceRNA regulatory network can provide reference targets for finding targets to maintain skeletal muscle function and treat muscle-related diseases. On the other hand, it also provides a reference for producing more marble-like meat products in animal husbandry production.

## Figures and Tables

**Table 1:** Primers used for real-time qPCR assays.

Gene ID	Gene Name	Accession Number	Forward Primer	Reverse Primer	Product Size
*CD36*	CD36 molecule	NM_001044622.1	GTTCTCAATCTGGCTGTG	CTGTGGTAGGAATAGGGT	184 bp
*ADIPOQ*	adiponectin, C1Q and collagen domain containing	NM_214370.2	TATGATGTCACCACTGGCAAA	TAGAGGAGCACAGAGCCAGAG	185 bp
*MYH2*	myosin, heavy chain 2, skeletal muscle, adult	NM_214136.1	TCTCAGGCTTCAGGATTTG	GCTTGCGGAATTTAGATAGA	113 bp
*SLC25A4*	solute carrier family 25 member 4	XM_003133345.4	CATTGATTGCGTGGTGAG	CGTTGAAACCCTGGTAGAG	374 bp
*PDK4*	pyruvate dehydrogenase kinase 4	NM_001159306.1	GACCCTTGGGACACTTTA	CATTGACTTCTGCCATCC	193 bp
*HADHB*	hydroxyacyl-CoA dehydrogenase trifunctional multienzyme complex subunit beta	NM_001348972.1	CTGGGTCAGCGACTGTCTTT	CGTACTCATCCTGCTCCGAC	160 bp
*COQ8A*	coenzyme Q8A	XM_021064416.1	CCGTCCTCTGAAATGACTCTTG	TGGTGAGGTCGTATTTGTTGG	295 bp
*ACADM*	acyl-CoA dehydrogenase medium chain	NM_214039.1	CCCCTTATTATTGGTGGA	TGCTTTGGTCTTTATCCC	135 bp
*THRSP*	thyroid hormone responsive	NM_001244376.1	AGGAGGTGACGAGGAAAT	CTCAGAGGAAGGGAAGGA	198 bp
*CPT1B*	carnitine palmitoyltransferase 1B	NM_001007191.1	AGGCCTCATCAAGAAGTGCC	TGAACGAAGGCTGTGGACTC	183 bp

## Data Availability

All relevant data are contained within the manuscript.

## Supplementary Materials

The following supporting information can be downloaded at https://www.mdpi.com/article/10.3390/cimb48050463/s1.
