# Supplementary material for: Characterization of Genes Related to Intramuscular Fat Deposition in Muscles of Piglets Under Cold Exposure by Whole Transcriptome Sequencing
Source: Curr Issues Mol Biol. 2026 Apr 29;48(5):463. doi: 10.3390/cimb48050463 (PMC13205075; doi:10.3390/cimb48050463)
Supplement: Supplementary file 1 [file cimb-48-00463-s001.zip › Supplementary Figure S1.pdf]

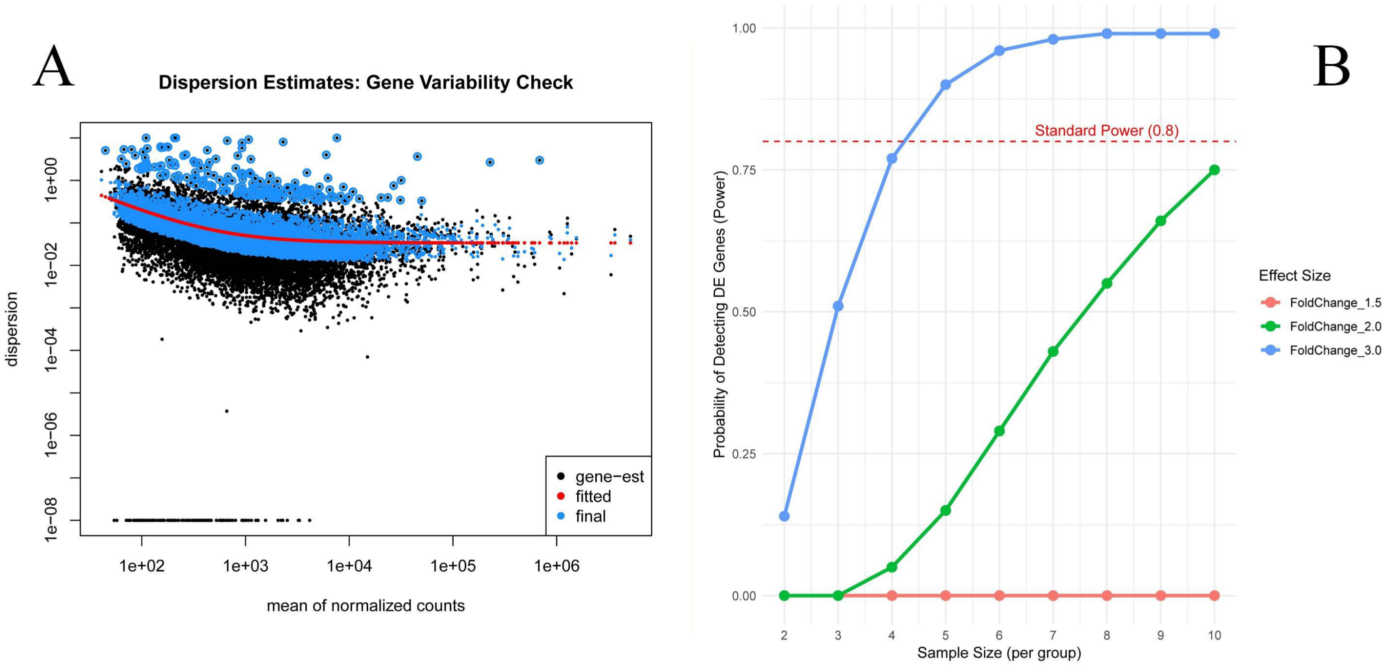

Supplementary Figure S1. Dispersion estimates and power calculations of mRNA data. (**A**) Dispersion plot of mRNA estimates. Gene-wise estimates (black), the fitted values (red), and the final maximum a posteriori estimates used in testing (blue). The blue circle and black heart represent outliers, which are allowed to appear in small quantities. (**B**) Power calculations of mRNA.
